# Prognostic performance of endothelial biomarkers to early predict clinical deterioration of patients with suspected bacterial infection and sepsis admitted to the emergency department

**DOI:** 10.1186/s13613-020-00729-w

**Published:** 2020-08-12

**Authors:** Thomas Lafon, Marie-Angélique Cazalis, Christine Vallejo, Karim Tazarourte, Sophie Blein, Alexandre Pachot, Pierre-François Laterre, Said Laribi, Bruno François, the TRIAGE study group, Julien Textoris, Karine Kaiser, Laurence Barbier, Laurent Jacquin, Marion Douplat, Amélie Nicol, Marine Delaroche, Landry Jacquet, Nathalie Chapelle-Petit, Abdel Chekaoui, Franck Verschuren, Manon Fabry, Valérie Gissot, Julie Magnan, Thomas Daix, Arnaud Desachy, Philippe Vignon, Marine Goudelin, Bruno Evrard, Anne Laure Fedou, Thibault Desmettre, François Jardin, Anne Claire Montini, Anaïs Colonna, Lionel Bertrand, Maxime Maignan, Damien Viglino, Mustapha Sebbane, Jacques Remize, Caroline Anoot, Jérome Frey, Agathe Pancher, Khalil Takun, Florence Dumas, Yves Lambert, Mehrsa Koukabi, Sabrina Measso, Ludovic Dalle, Olivier Dupeux, Antoine Duconge

**Affiliations:** 1 Emergency Department, Dupuytren University Hospital, Limoges, France; 2 Inserm CIC 1435, Dupuytren University Hospital, Limoges, France; 3 Medical Diagnostic Discovery Department MD3, bioMerieux SA, Marcy L’Etoile, France; 4 Emergency Department, University Hospital Edouard Herriot – HCL, Lyon, France; 5 Departments of Emergency and Intensive Care, Cliniques Universitaires Saint Luc, UCL, Brussels, Belgium; 6 School of Medicine and Tours University Hospital, Emergency Medicine Department, Tours University, Tours, France; 7 Medical-Surgical Intensive Care Unit, Dupuytren University Hospital, Limoges, France; 8 UMR 1092, University of Limoges, Limoges, France

**Keywords:** Sepsis, Clinical deterioration, Biomarkers, Prognosis, Endothelium, Emergency medicine

## Abstract

**Background:**

The objective of this study was to evaluate the ability of endothelial biomarkers to early predict clinical deterioration of patients admitted to the emergency department (ED) with a suspected sepsis. This was a prospective, multicentre, international study conducted in EDs. Adult patients with suspected acute bacterial infection and sepsis were enrolled but only those with confirmed infection were analysed. The kinetics of biomarkers and organ dysfunction were collected at T0, T6 and T24 hours after ED admission to assess prognostic performances of sVEGFR2, suPAR and procalcitonin (PCT). The primary outcome was the deterioration within 72 h and was defined as a composite of relevant outcomes such as death, intensive care unit admission and/or SOFA score increase validated by an independent adjudication committee.

**Results:**

After adjudication of 602 patients, 462 were analysed including 124 who deteriorated (27%). On admission, those who deteriorated were significantly older (73 [60–82] vs 63 [45–78] y-o, *p* < *0.001*) and presented significantly higher SOFA scores (2.15 ± 1.61 vs 1.56 ± 1.40, *p* = *0.003*). At T0, sVEGFR2 (5794 [5026–6788] vs 6681 [5516–8059], *p* < *0.0001*), suPAR (6.04 [4.42–8.85] vs 4.68 [3.50–6.43], *p* < *0.0001*) and PCT (7.8 ± 25.0 vs 5.4 ± 17.9 ng/mL, *p* = *0.001*) were associated with clinical deterioration. In multivariate analysis, low sVEGFR2 expression and high suPAR and PCT levels were significantly associated with early deterioration, independently of confounding parameters (sVEGFR2, OR = 1.53 [1.07–2.23], *p* < *0.001*; suPAR, OR = 1.57 [1.21–2.07], *p* = *0.003*; PCT, OR = 1.10 [1.04–1.17], *p* = *0.0019*). Combination of sVEGFR2 and suPAR had the best prognostic performance (AUC = 0.7 [0.65–0.75]) compared to clinical or biological variables.

**Conclusions:**

sVEGFR2, either alone or combined with suPAR, seems of interest to predict deterioration of patients with suspected bacterial acute infection upon ED admission and could help front-line physicians in the triage process.

## Background

Sepsis is a major health burden with considerable economic consequences [1]. Recently, epidemiological studies performed on large cohorts reported that sepsis was present in 6% of adult hospitalizations [2]. Over the last decade, a decrease in the mortality rate has been observed [3] in particular thanks to improved management, and more appropriate intervention approaches in the emergency department (ED) [4]. Although the recently proposed qSOFA score [5] aims to help front-line clinicians detecting severe patients with a higher risk of mortality [6], it fails to get decisive support for discharge decision, especially in patients without initial organ dysfunction [7, 8], that could help to reduce ED crowding and cost.

Even if widely used as an infection biomarker and diagnostic of severity, procalcitonin (PCT) has not been fully validated for deterioration assessment, and no other biological marker has yet been validated to accurately early predict clinical deterioration in unselected patients admitted to the ED with infection or sepsis [9–11]. Asymptomatic endothelial injury participates in the development of organ failure with poor outcome [12, 13]. Endothelial biomarkers have been presented as predictors of death and/or organ dysfunction during sepsis [14–22]. Of those, soluble vascular endothelial growth factor receptors 2 (sVEGFR2, growth factor for vascular endothelial cells) and soluble urokinase plasminogen activator receptor (suPAR, pro-inflammatory activation of the immune system) were proposed. VEGFR2, which is selectively expressed in the endothelium, mediates endothelial growth, proliferation and permeability and pathological angiogenesis, and bound to VEGF increases microvascular permeability resulting in oedema and hypotension [23]. The uPAR receptor is expressed on different cell types including vascular endothelial cells [24]. After cleavage from the cell surface, the soluble receptor, suPAR, can be found in the blood and other organic fluids. Increased activation of the immune system caused by different types of infections results in increased suPAR concentrations. These biomarkers have been shown to be associated with initial severity and subsequent clinical worsening [25–32], but their ability to early predict deterioration on ED admission remains to be determined.

This study aimed to evaluate the ability of sVEGFR2 and suPAR biomarkers to early predict the clinical deterioration of patients with infection upon ED admission and compare them to conventional clinical and biological parameters (qSOFA and SOFA score, lactates, PCT, CRP). Second, we assessed the prognostic performance of biomarkers according to the presence of sepsis or not in accordance with the new definitions of Sepsis-3.

## Methods

### Population

We conducted a prospective, multicentre, international study in 14 EDs from 2015 to 2018. Inclusion criteria included adult patients (age ≥ 18 years) with an acute suspected bacterial community-acquired infection (≤ 3 days), evolution time window being checked with the patient and/or relatives, associated with at least two systemic inflammatory response syndrome (SIRS) criteria [33], which were currently the most sensitive criteria for sepsis [34, 35]. All patients admitted to the ED with a suspected infection, based on fever and/or any other infectious symptom reported by referral practitioner were screened 24/7 by emergency physicians for eligibility and treated following the *Surviving Sepsis Campaign* guidelines [36]. Exclusion criteria were patients with septic shock (based on ACCP/SCCM criteria), patients with a healthcare-associated infection, immunosuppression (e.g. human immunodeficiency virus (HIV), transplant, ongoing chemotherapy, steroid treatment > 20 mg/day of prednisone or equivalent for more than a week), non-infectious diseases potentially associated with SIRS (cancer), patients with a prior episode of infection within the 30 days before ED admission and onset of symptoms greater than 72 h and absence of consent. The protocol was recorded on ClinicalTrials.gov (N°: NCT02739152), approved by the Ethics Committee for Clinical Research (CPP SOOM IV: CPP15-004).

### Endpoints

The primary endpoint was the occurrence of early clinical deterioration within 72 h following ED admission. Deterioration was determined by an independent adjudication committee (including one experienced emergency physician and two intensive care physicians) who were blinded to biomarker results, and followed a pre-defined adjudication charter. Patients were then classified according to their initial course during the first 72 h of hospitalization, as exhibiting an early deterioration defined by a composite endpoint (increase SOFA score of at least 1 point, or ICU admission directly related to the initial infectious disease because of documented sustained hypotension requiring vasopressors or ventilation support requirement, or death) or not. The same adjudication committee also confirmed the bacterial origin of infection according to available clinical, biological and microbiological data and based on pre-defined criteria for every different type of infection [37]. Patients without confirmed infection were excluded from the analysis.

### Study design and measured variables

All the patients were included and received their first care and blood collection in ED. Clinical criteria, biological data, lactates [38], qSOFA score, SOFA score [39] and studied biomarkers were measured at three time points: the first within emergency room (T0) and the others at 6 ± 2 h (T6) and 24 ± 2 h (T24) after ED admission. The following data were prospectively collected by the study team blinded to biomarker results during ED stage: demographics, Charlson score, site of infection, antimicrobial therapy and initiation time, traditional biological parameters (leukocytes, CRP, platelets) and orientation after ED discharge. Pathogens, length of stay and mortality at day 28 were collected during hospitalization or at the end of follow-up.

### Biomarker measurements

Serum sVEGFR2 concentrations (soluble vascular endothelial growth factor receptors 2) were measured using the enzyme-linked fluorescent assay (ELFA) technique. The results were automatically analysed by VIDAS® and expressed in relative fluorescence intensity or RFV (relative fluorescent value). Plasma suPAR (soluble urokinase plasminogen activator receptor) levels were analysed using the commercially available CE/IVD-labelled suPARnostic® AUTO Flex ELISA kit, according to the manufacturer’s instructions (Virogates, Birkeroed, Denmark). For sUPAR ELISA test, the inter-assay coefficient of variation (CV) given by the manufacturer is below 6%. For sVEGFR2, the inter-assay coefficient of variation was calculated at 3.09%. Serum PCT (procalcitonin) levels were measured using VIDAS BRAHMS PCT assay (Biomerieux, Marcy l’Etoile, France) according to the manufacturer’s instructions.

### Analysis

The prognostic performance of studied biomarkers was evaluated in the entire cohort at T0. Following the current definitions and to analyse the prognostic performance of biomarkers according to the severity, the Sepsis-3 criteria were applied to define 2 groups: infected patients (SOFA score < 2) and septic patients (SOFA score ≥ 2) [5]. A model of risk of early deterioration based on the value of biomarkers on ED admission was proposed on non-septic patients. Data were censored after deterioration. No patient was lost to follow-up until T24.

### Statistics

Data are presented either as means ± SD, median with interquartile range or as box and whisker plots with representation of the median value, 25th, 75th and 90th percentiles, and outliers. Parameters and biomarkers were compared between the two groups of patients according to their initial course (i.e., early deterioration or not), using the nonparametric Mann–Whitney *U* test for continuous variables, while categorical variables were compared with the Pearson *χ*^2^ test or the Fisher’s exact test when appropriate. The level of significance was set at 5% and results of regression analyses were presented with their 95% CI. All analyses were computed using the R version 3.4.0.

Logistic regressions were fit using single or both biomarkers. Association with clinical variables was independently evaluated, and clinical parameters with a *p *value below 0.1 in the univariate analyses were selected as adjustment covariates for multivariate analyses. Among significant clinical parameters, a selection was made to avoid collinearity and limit the number of variables introduced in multivariate models. Strength of association was reported using inter-quartile range (IQR) adjusted odds ratios (OR). Areas under the ROC curve and their 95% confidence interval (CI) were computed and compared using the DeLong’s method. Predictive performances were calculated/evaluated under constraint of a sensitivity higher than 90% (rule-out test). In a complementary approach, a decision tree was built. The thresholds used to partition the data were chosen to optimize sensitivity (> 90%).

## Results

### Study population

**Fig. 1:**
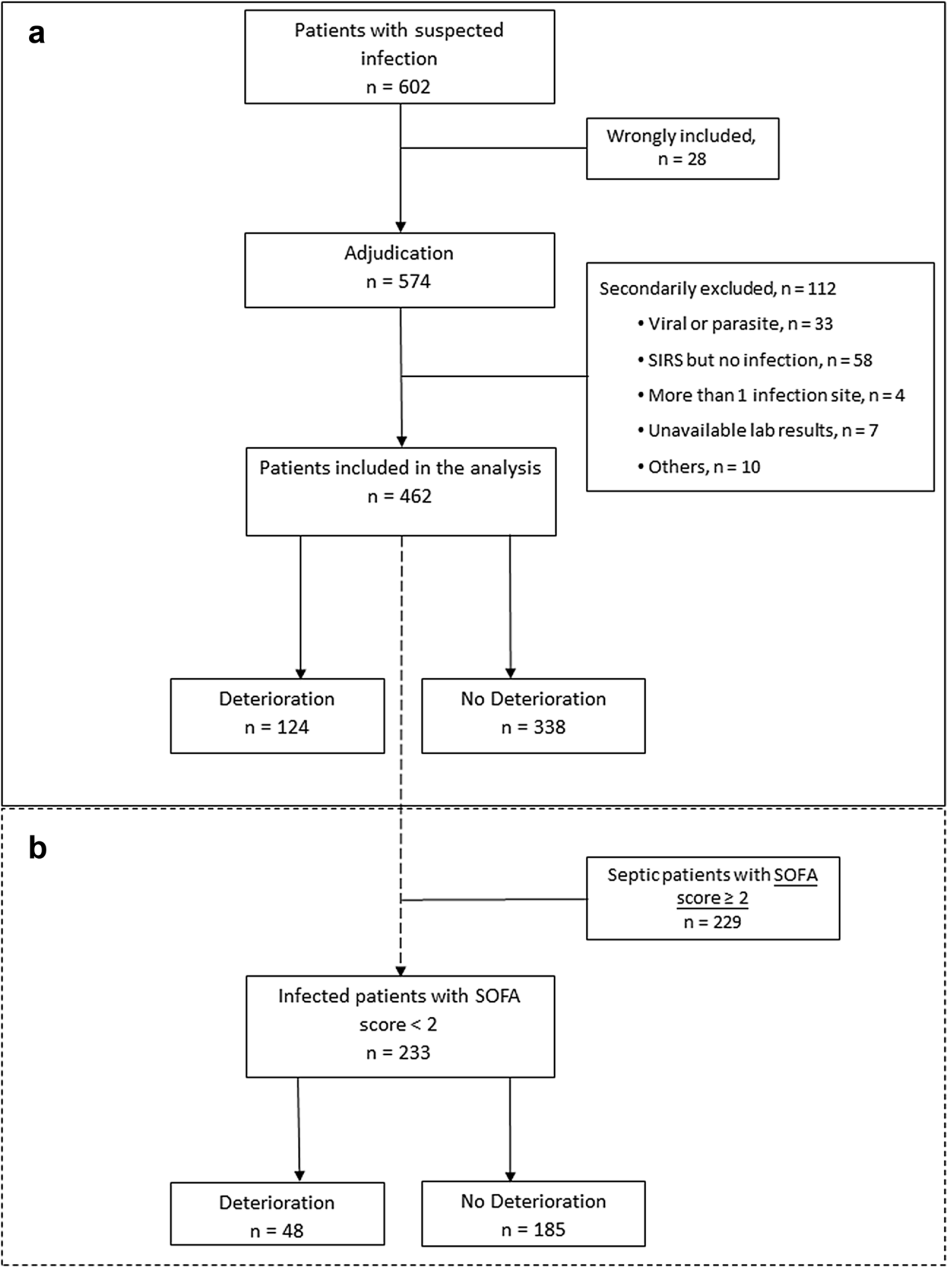
Study flowchart. **a** A total of 602 patients admitted to the emergency department and suspected of bacterial infection were enrolled. After adjudication, 462 patients were included in the analysis. **b** Infected non-septic patients according to Sepsis-3 definition; SOFA under 2 point at admission to the emergency department

Of 602 patients enrolled in the 14 participating EDs between 2015 and 2018, 28 patients were secondarily excluded due to the presence of concomitant cancer potentially responsible for SIRS. Independent adjudication was performed in 574 patients, out of whom 112 patients were classified without bacterial infection nor confirmed infection (10%). Finally, 462 patients with a bacterial acute community-acquired infection were kept for the analysis (Fig. 1a). Baseline characteristics are described in Table 1. Infections were mainly of pulmonary (29.2%), urinary (27.5%) and abdominopelvic (25.3%) origin. Microorganisms were isolated from 51% of patients (cocci = 13%, bacilli = 34% and both = 4%). At baseline, the median lactatemia was 1.7 [1.2–2.4] mmol/L, median leukocyte was 14.6 [11–18] G/L and median CRP was 115 [43–219] mg/L (Table 1). Median time of antibiotic treatment was 4.1 h [2.3–6.7]. After ED management, 87% of patients were hospitalized in a conventional ward while the remaining 13% were admitted to the ICU. Median hospital stay was 5 [3–9] days and 28-day mortality was 2.6% (Table 1). Mean SOFA score was 1.72 ± 1.48. Based on the new Sepsis-3 definition, 229 patients (49.6%) had sepsis (SOFA ≥ 2 points) while the remaining 233 patients (50.4%) had infection (SOFA < 2 points) (Fig. 1b).

**Table 1:** Clinical and demographic characteristics of the study population (*n* = 462)

	Deterioration (*n* = 124)	No deterioration (*n* = 338)	Total (*n *= 462)	*p* value
Male	70 (56)	182 (54)	252 (55)	0.694^a^
Age (years)	73 [60–82]	63 [45–78]	66 [48–80]	< .001^b^
Numbers of comorbidities				0.001^a^
0	46 (37)	188 (56)	234 (51)	
1	38 (31)	84 (25)	122 (26)	
> 1	40 (32)	66 (20)	106 (23)	
Comorbidities, *n* (%)				
Cardiovascular disease	31 (25)	36 (11)	67 (15)	< .001^a^
Renal disease	11 (9)	9 (3)	20 (4)	0.003^a^
Respiratory disease	27 (22)	45 (13)	72 (16)	0.023^a^
Diabetes	33 (27)	77 (23)	110 (24)	0.39^a^
Charlson score	4 [2.75–5]	2 [0–5]	3 [1–5]	< .001^b^
Diagnostic category at T0				1.000^a^
Sepsis	85 (69)	232 (69)	317 (69)	
Severe sepsis	39 (31)	106 (31)	145 (31)	
SOFA category at inclusion				0.003^a^
SOFA < 2	48 (39)	185 (55)	233 (50)	
SOFA ≥ 2	76 (61)	153 (45)	229 (50)	
Lactates (mmol/L)	1.7 [1.2–2.3]	1.7 [1.2–2.5]	1.7 [1.2–2.4]	0.999^b^
Lymphocytes (G/L)	0.78 [0.4–1.1]	0.94 [0.6–1.4]	0.9 [0.5–1.3]	0.003^b^
qSOFA score				0.018^c^
0	39 (31)	14 (44)	188 (41)	
1	81 (65)	185 (55)	266 (58)	
2	4 (3)	4 (1)	8 (2)	
Site of infection				0.049^a^
Pulmonary	46 (37)	89 (26)	135 (29)	
Urinary	25 (20)	102 (30)	127 (27)	
Abdominopelvic	34 (27)	83 (25)	117 (25)	
Others	19 (15)	64 (19)	83 (18)	
Hospitalization after ED discharge				< .001^a^
Ward	74 (60)	209 (64)	283 (63)	
Surgery	21 (17)	87 (27)	108 (24)	
Stepdown unit	12 (10)	21 (6)	33 (7)	
ICU	16 (13)	9 (3)	25 (6)	
ATB delay (hours)	3.9 [2.4–7]	4.1 [2.3–6.7]	4.1 [2.3–6.7]	0.675^b^
LOS (days)	8 [6–13]	5 [3–7]	5 [3–9]	< .001^b^
D-28 mortality	6 (5)	6 (2)	12 (3)	0.132^a^

Data are expressed either as* n* (%) or as median [Q1, Q3]

*ATB* antibiotherapy, *ICU* intensive care unit, *LOS* length of stay

^a^Pearson *χ*^2^ test

^b^Wilcoxon–Mann–Whitney

^c^Fisher

### Deterioration group

Among the 462 analysed patients, 124 patients (27%) were assigned to the “deterioration” group by the adjudication committee. Deterioration occurred within the first 6 h of inclusion for 64% of them. Thirty-nine patients progressed to severe sepsis and 11 patients to septic shock [32]. When compared with the “no deterioration” group (*n* = 338 patients, 73%), patients in the “deterioration” group were older (73 [60–82] vs 63 [45–78] years, *p* < *0.001*), had a higher Charlson score (4 [2.75–5] vs 2 [0–5], *p* < *0.001*), more frequent pulmonary infections (37.1 vs 26.3%, *p* = *0.049*) and greater SOFA score (2.15 ± 1.61 vs 1.56 ± 1.40, *p* < *0.001)*. Patients with early deterioration were more frequently admitted to ICU (13 vs 3%, *p* < *0.001*) and had a higher length of stay (8 [6–13] vs 5 [3–7] days, *p* < *02.001*). In contrast, there was no statistical difference between the two groups on SIRS criteria count (3.0 ± 0.7 vs 2.9 ± 0.7, *p* = *0.073*), lactates (2.04 ± 1.35 vs 2.09 ± 1.41 mmol/L, *p* = *0.999*), CRP level (160 ± 127 vs 141 ± 126 mg/L, *p* = *0.087*) and time to antibiotic treatment initiation (5.3 ± 4.8 vs 6.1 ± 7.5 h, *p* = *0.675*) (Table 1). Interestingly, we did not find any difference between the groups “deterioration” and “no deterioration” regarding initial adequate antibacterial treatment (20 vs 15%, *p* = *0.246*).

### Biomarkers: predictive performances in the global cohort

At T0 in univariate analysis, the age, the Charlson score, the qSOFA score and the SOFA score were associated with the early clinical deterioration, but not traditional biological markers. On the global cohort, sVEGFR2 and suPAR level were, respectively, 6402 [5386–7715] ng/mL and 5.00 [3.67–6.86] ng/mL. Low sVEGFR2 level (5794 [5026–6788] vs 6681 [5516–8059], *p* < *0.0001*), high circulating suPAR (6.04 [4.42–8.85] vs 4.68 [3.50–6.43], *p* < *0.0001*) and high PCT level (7.8 ± 25.0 vs 5.4 ± 17.9 ng/mL, *p* = *0.001*) were associated with early clinical deterioration (Table 2). In multivariate logistic regression analyses, only low sVEGFR2 expression, high suPAR and PCT levels were significantly associated with deterioration (sVEGFR2: OR [95% CI] = 1.53 [1.07–2.23], *p* < *0.001*; suPAR: OR = 1.57 [1.21–2.07], *p* = *0.003* and PCT: OR = 1.10 [1.04–1.17], *p* = *0.0019*), independently of covariates (Charlson, qSOFA, age, SOFA). The sVEGFR2 and sUPAR combination showed the higher predictive performances compared to other variable (AUC = 0.70 [0.65–0.75]) (Table 2). We also observed differences regarding site of infections, with the best performances found in abdominal infections (AUC = 0.81 [0.73–0.89], NPV 93%), then urinary (AUC = 0.7 [0.59–0.80], NPV = 95%) and finally pulmonary (AUC = 0.66 [0.56–0.76], NPV = 79%).

**Table 2:** Factors predictive of early deterioration using univariate and multivariate analyses in the entire cohort (*n* = 462) at inclusion (T0)

Variable	Univariate analysis		Multivariate analysis		Predictive performance			
	*p* value	IQR OR [95% CI]	*p* value	IQR OR [CI]	AUC [CI]	sp	NPV	PPV
Lactates	ns	–	ns	–	0.50 [0.43–0.57]	0.12	0.76	0.29
CRP	ns	–	ns	–	0.55 [0.49–0.61]	0.16	0.81	0.28
qSOFA score	7.89E−03	1.73 [1.16–2.61]	ns	–	0.57 [0.52–0.62]	0.14	0.79	0.28
Lymphocytes	3.00E−03	1.45 [1.19–2.17]	ns	–	0.59 [0.54–0.66]	0.18	0.86	0.28
SOFA score	2.27E−04	1.29 [1.13–1.49]	ns	–	0.61 [0.55–0.66]	0.18	0.83	0.29
Age	9.55E−05	1.25 [1.12–1.40]	ns	–	0.62 [0.57–0.68]	0.21	0.85	0.29
Charlson score	2.43E−05	1.19 [1.10–1.29]	ns	–	0.64 [0.59–0.69]	0.23	0.86	0.3
PCT	1.35E−04	1.11 [1.05–1.17]	1.90E−03	1.10 [1.04–1.17]	0.62 [0.56–0.68]	0.16	0.82	0.28
suPAR	2.28E−08	1.92 [1.53–2.42]	3.16E−03	1.57 [1.21–2.07]	0.66 [0.60–0.72]	0.20	0.84	0.29
sVEGFR2	7.19E−06	2.11 [1.53–2.94]	6.59E−04	1.53 [1.07–2.23]	0.65 [0.60–0.71]	0.25	0.87	0.31
suPAR–sVEGFR2 Combination	1.32E−09	2.14 [1.69–2.76]	4.38E−04	1.78 [1.30–2.47]	0.70 [0.65–0.75]	0.33	0.90	0.33

*CRP* C-reactive protein, *SOFA* Sequential Organ Failure Assessment, *qSOFA* quick SOFA, *PCT* procalcitonin, *IQR* interquartile, *OR* odds ratio, *CI* confidence interval, *Sp* specificity calculated for a sensitivity higher than 0.90, *NPV* negative predictive value, *PPV* positive predictive value

Biomarker association with clinical deterioration was also observed in secondary excluded patients (Additional file 1: Figure 1).

**Fig. 2:**
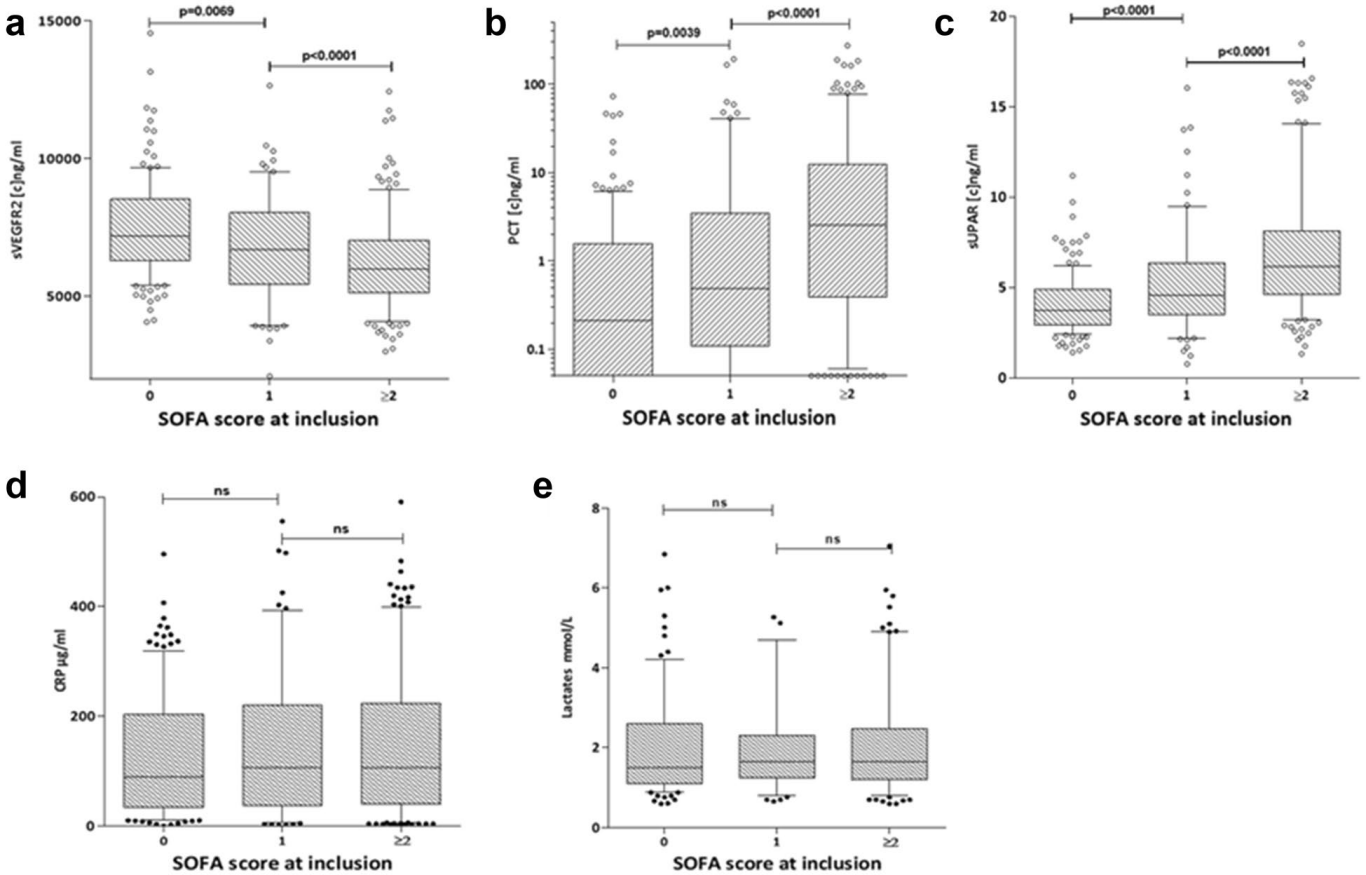
Relationship between initial organ failure (SOFA at inclusion) and expression levels of sVEGFR2 (**a**), PCT (**b**), suPAR (**c**), CRP (**d**) and lactates (**e**) measured at inclusion (T0). Statistically significant differences (Mann–Whitney *U* test) are observed for the first 3 groups

Levels of sVEGFR2, PCT and suPAR at inclusion were significantly associated with the degree of organ dysfunction, as reflected by the SOFA score on ED admission (Fig. 2).

### Biomarkers: predictive performances in infected patients without sepsis (SOFA < 2)

Among the 233 patients considered with infection but no sepsis at enrolment (SOFA < 2 at admission), 48 of them (21%) deteriorated within 72 h (Fig. 1b). The clinical characteristics of this derivative cohort at admission were not different from those of the overall cohort (Table 3). In univariate logistic regression analyses, age and Charlson score were significantly associated with deterioration. Low sVEGFR2 expression and high suPAR level, either taken alone or combined, were also significantly associated with deterioration (sVEGFR2, IQR OR [95% CI] = 2.76 [1.68–4.75], *p* = *0.0002*; suPAR IQR OR = 1.49 [1.06–2.10], *p* = *0.022* and combination IQR OR = 1.74 [1.28–2.41], *p* = *0.0005*). In multivariate analysis, only sVEGFR2 alone or combined with suPAR remained significantly associated with deterioration (IQR OR [95% CI] = 2.35 [1.41–4.12], *p* = *0.0017* and IQR = 1.52 [1.07–2.19], *p* = *0.019*, respectively) (Table 4). At T6, only sVEGFR2 and sUPAR were found significantly associated with worsening (*p* < *0.01*) (Additional file 1: Figure 2b.). As data were censored after deterioration, the low number of patients deteriorating after T6 and T24 did not allow further analysis. The prognostic value of biomarkers on septic patients (SOFA ≥ 2) is presented in Additional file 1: Table 1.

**Table 3:** Clinical and demographic characteristics of infected non-septic patients according to clinical course during the 72 h after admission to the emergency department

Variable	Deterioration (*n* = 48)	No deterioration (*n* = 185)	Total (*n* = 233)	*p* value
Male	24 (50.00)	87 (47.03)	111 (47.64)	0.837^a^
Age (years)	66 [42–73]	52 [35–67]	55 [36–69]	0.004^b^
Number of comorbidities				0.132^a^
0	26 (54.17)	128 (69.19)	154 (66.09)	
1	15 (31.25)	36 (19.46)	51 (21.89)	
> 1	7 (14.58)	21 (11.35)	28 (12.02)	
Charlson score	3 [0.75–5]	1 [0–3]	1 [0–3]	0.001^b^
qSOFA				0.357^a^
0	22 (45.83)	101 (54.59)	123 (52.79)	
1	26 (54.17)	84 (45.41)	110 (47.21)	
Site of infection				0.132^a^
Pulmonary	14 (29.17)	27 (14.59)	41 (17.60)	
Urinary	13 (27.08)	62 (33.52)	75 (32.19)	
Abdominopelvic	13 (27.08)	58 (31.35)	71 (30.47)	
Others	8 (16.67)	38 (20.54)	46 (19.74)	
Hospitalization after ED discharge				0.758^c^
Ward	29 (61.69)	100 (56.82)	129 (57.84)	
Surgery	14 (29.79)	64 (36.36)	78 (34.98)	
Stepdown unit	2 (4.26)	7 ( 3.98)	9 (4.04)	
ICU	2 (4.26)	5 ( 2.84)	7 (3.14)	
ATB delay (hours)	5.08 [3.4–8.0]	4.34 [2.6–7.0]	4.60 [2.7–7.6]	0.109^b^
LOS (days)	6.5 [3.8–10]	4 [3–6]	4 [3–7]	< .001^b^
D-28 mortality	1 (2.08)	2 (1.08)	3 (1.29)	0.501^c^

Data are expressed either as n (%) or as median [Q1, Q3]

*ATB* antibiotherapy, *ICU* intensive care unit, *LOS* length of stay

^a^ Pearson *χ*^2^ test

^b^ Wilcoxon–Mann–Whitney

^c^ Fisher

**Table 4:** Factors predictive of early deterioration using univariate and multivariate analyses in non-septic infected patients (SOFA score < 2) at inclusion (T0) (*n* = 223)

Variable	Univariate analysis		Multivariate analysis	
	*p* value	IQR OR [95% CI]	*p* value	IQR OR [95% CI]
Lactates	ns	–	ns	–
CRP	ns	–	ns	–
qSOFA score	ns	–	ns	–
Age	0.0042	1.27 [1.08–1.51]	ns	–
Charlson score	0.0029	1.25 [1.08–1.44]	ns	–
PCT	ns	–	ns	–
sUPAR	0.022	1.49 [1.06–2.10]	ns	–
sVEGFR2	0.0002	2.76 [1.68–4.75]	0.0017	2.35 [1.41–4.12]
sUPAR -sVEGFR2 combination	0.0005	1.74 [1.28–2.41]	0.019	1.52 [1.07–2.19]

*CRP* C-reactive protein, *qSOFA* quick Sequential Organ Failure Assessment, *PCT* procalcitonin, *IQR* interquartile, *OR* odds ratio, *CI* confidence interval

### Proposal of a stratification model in non-severe patients on ED admission

**Fig. 3:**
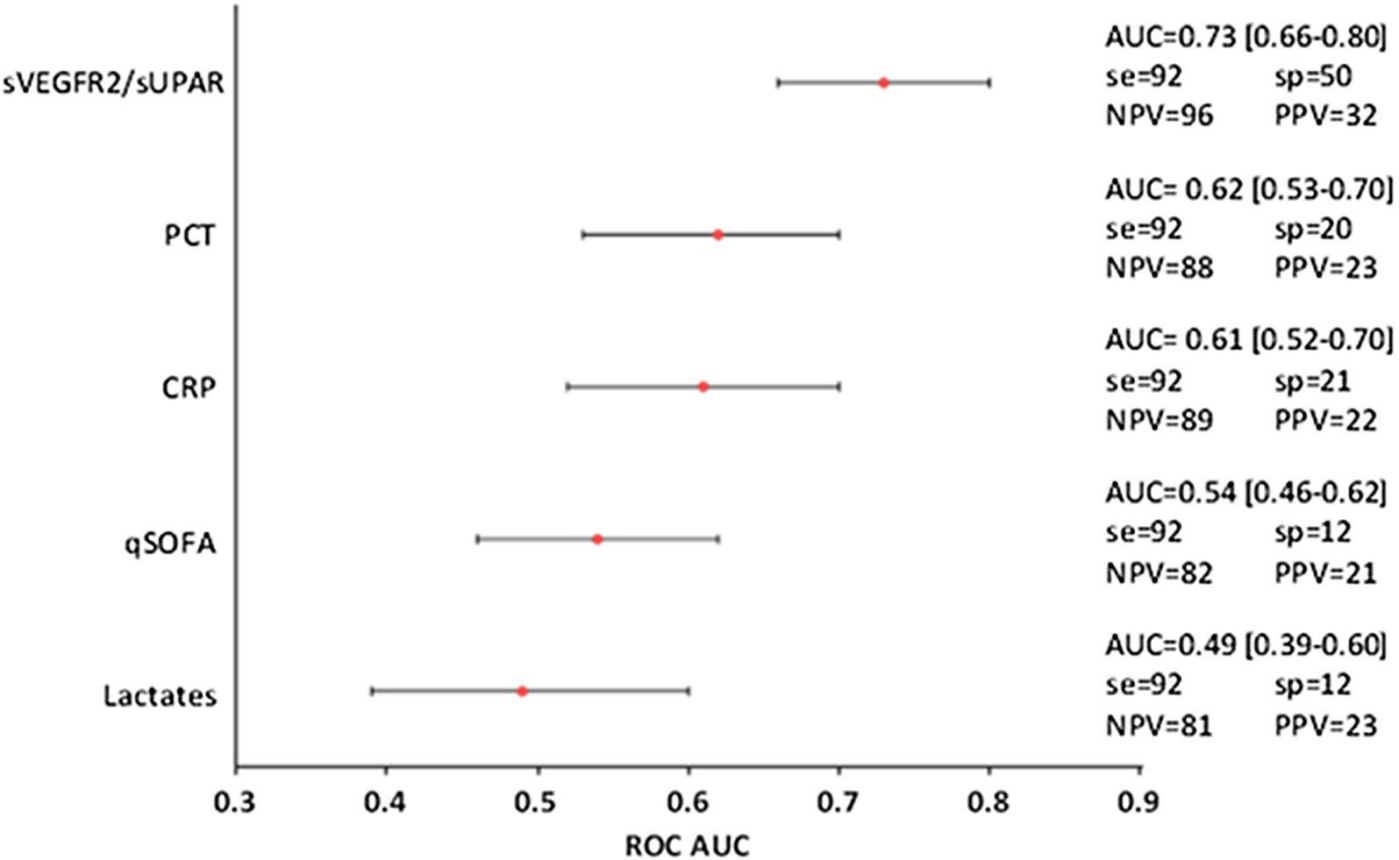
Comparison of predictive performance of biomarkers measured at inclusion (T0) on non-severe-infected patients. Sensitivity, specificity and predictive values of biomarkers according to optimized cut-offs to minimal sensitivity of 90%. Abbreviations: *AUC [IC]* area under the curve and confidence interval (two-sided *p* value < 0.05), *se* sensitivity (%), *sp* specificity (%), *NPV* negative predictive value (%), *PPV* positive predictive value (%)

The best prognostic model including sVEGFR2 and suPAR combination and using cut-off values optimized to yield a high sensitivity allowed identifying distinct levels of risk (i.e. low and high) for deterioration. When comparing risk groups, we found that the low-risk group had a 15-fold lower risk of worsening than the high-risk group (OR = 14.50 [4.97–61.85]; *p* < *0.0001*) (Additional file 1: Figure 3). This model proved a modest clinical deterioration performance (AUC = 0.73 [0.66–0.80]; *p* < *0.0001*) with a promising negative predicting value (96%). The AUC of this model was significantly higher than that of lactate (AUC = 0.49 [0.39–0.60]; *p* > *0.05*), qSOFA score (AUC = 0.54 [0.46–0.62]; *p* > *0.05*), CRP (AUC = 0.61 [0.52–0.70]; *p* < *0.05*), and PCT (AUC = 0.62 [0.53–0.70]; *p* < *0.05*) (Fig. 3).

## Discussion

In this ED-based multicentre study, the endothelial biomarker sVEGFR2, used either alone or combined with suPAR, proved the best early predictor of patient deterioration, independently of potential confounders. High medical value biomarkers are those that are able to predict outcomes even before any clinical evidence of deterioration to help front-line physicians to better anticipate the complicated course. Since early prediction of patient deterioration is crucial to allow safe rule-out, over-triage reduction and better allocation of hospital resources, the high negative value of these potential endothelial biomarkers appears particularly useful in the ED settings with inherent peaks of activity and overcrowded units.

In the present study, 27% of the entire cohort deteriorated within 72 h of ED admission. Importantly, 21% of non-sepsis patients, without any severity criteria on ED admission, did deteriorate within the first 72 h of hospitalization. Intrinsically, these patients presented to the ED with low SOFA (i.e., SOFA < 2) and non-qualifying qSOFA score. This proportion is similar to that reported in previous studies [18–20]. About 20 to 25% of patients progressed to severe sepsis whereas they had no sign of seriousness at first medical contact [7]. Saeed et al. reported that early clinical deterioration occurred in more than 16% of patients presenting to the ED with sepsis, even when patients were non-severe with low lactate level (< 2 mmol/L) or low clinical score (qSOFA < 2) [40]. Recently, Cleek et al. confirmed that in predicting 28-day in-hospital mortality among infected ED patients, qSOFA did not outperform or improve physician judgment [41]. Overall, the clinical deterioration occurred very early after ED admission since two-third of the patients deteriorated within the first 6 h of inclusion. Although information on the delay between ED admission and deterioration are scare, some authors have reported that it may occur within 48 h [42], even within the first 12 h following ED arrival [43].

Due to various presentations of infected patients on ED admission, determining the severity early in the disease course remains challenging since clinical scoring systems have limited prognostic accuracy [44, 45]. Many conventional biomarkers reflecting end-organ compromise are not informative until significant clinical deterioration has occurred [46, 47]. The Sepsis-3 definition underlines organ dysfunction as the mainstay of sepsis and the value of the SOFA score to identify patients with a higher risk of subsequent death [48]. However, 21% of our patients with a SOFA score < 2, i.e. non-sepsis according to Sepsis-3, deteriorated within 72 h after ED arrival. In these circumstances, assessment of endothelial injury could be a good predictor of deterioration [49]. Likewise Fang et al. [20] described a relationship between endothelial biomarkers and variations of the SOFA score during the first week after admission. Liu et al. [50] also showed an association between the presence of endothelial injury on admission and severity of sepsis. More recently, Henning et al. also confirmed that biomarkers of endothelial activation and inflammation in combination with emergency department physician judgment improved prediction in hospital mortality [51, 52]. These observations are concordant with our findings showing that the level of sVEGFR2 and suPAR is associated with that of the SOFA score. Importantly, we have shown that sVEGFR2 alone or combined with suPAR is the best predictor of patient deterioration, independently of potential confounding factors. If confirmed, this result could allow safe rule out of patients who have low risk of deterioration, hence leading to a decrease in hospital admissions.

This prospective, multicentre, international, observational study presents several strengths, such as (i) a biological collection of biomarkers combining with the evolving clinical criteria/in line with the requirements of the new Sepsis-3 definition of sepsis, and (ii) the appointment and careful evaluation by an independent adjudication committee. We also demonstrated that circulating markers of endothelial activation, at the earliest time in ED, have a potential of risk stratification and could help emergency physicians better manage patients with sepsis.

Our study, however, has several limitations, the first one being the limited possibility to fully investigate the heterogeneity of the different subtypes of infections. Indeed, patients with pneumonia may differ from patients with abdo-pelvic infections. The population may be biased against deterioration, as it requires decompensation from a less ill state. Half of the cohort having a SOFA ≥ 2 at ED baseline may have been already quite ill. Therefore, the study may have been stronger if focused on a light-/middle-severity sepsis cohort, using new definition of sepsis if it had been available when designing the study. Also, the design and purpose of the study did not allow analysis of patients with septic shock, while they could have been used as a control group of severity. In addition, as the number of patients with non-confirmed or viral infection was low, no prediction analysis was done. The entire analysis has somewhere a modest sample size (*n* = 462 patients, of which 127 experienced deterioration) and do not support strong conclusions but serve as a robust early basis for future validation. Finally, we did not perform health economics and outcome research that could have brought useful information on the potential cost savings for hospitals.

## Conclusion

The current findings highlight the potential interest of the sVEGFR2 protein, alone or in combination with suPAR, to diagnose initial endothelium stress and to predict/anticipate subsequent organ dysfunction. Such tool, suitable for routine test measurement, with time-to-results within 1 h and only one-time measurement required, could be used together with other laboratory findings and clinical assessments, to help in early prediction of the risk of deterioration and safely ruling out infected patients after ED admission.

## Supplementary information

**Additional file 1.** Additional figures and table.

## Abbreviations

AUC: Area under the curve
CI: Confidence interval
CRP: C-reactive protein
ED: Emergency department
ELFA: Enzyme-linked fluorescent assay
HIV: Human immunodeficiency virus
ICU: Intensive care unit
IQR: Inter-quartile range
OR: Odds ratio
PCT: Procalcitonin
RFV: Relative fluorescent value
SD: Standard deviation
SIRS: Systemic inflammatory response syndrome
SOFA: Sequential organ failure assessment
sUPAR: Soluble urokinase plasminogen activator receptor
sVEGFR2: Soluble vascular endothelial growth factor receptors 2

## Availability of data and materials

The datasets used and/or analysed during the current study are available from the corresponding author on reasonable request.
