# Supplementary material for: Prognostic performance of endothelial biomarkers to early predict clinical deterioration of patients with suspected bacterial infection and sepsis admitted to the emergency department
Source: Ann Intensive Care. 2020 Aug 12;10:113. doi: 10.1186/s13613-020-00729-w (PMC7423829; doi:10.1186/s13613-020-00729-w)
Supplement: Supplementary file 1 — Additional file 1. Additional figures and table. [file 13613_2020_729_MOESM1_ESM.pdf]

Supplemental data

Supplemental figure 1: Box plot of sVEGFR2 (A) and sUPAR (B) protein expression levels at inclusion (T0) in secondary excluded patients with or without deterioration. Secondary excluded patients are mainly SIRS without confirmed infection, viral infection, multi-infected patients, infected patients with cancer, etc. Box plot summarizes the median and interquartile ranges and P values < 0.05 are significant.

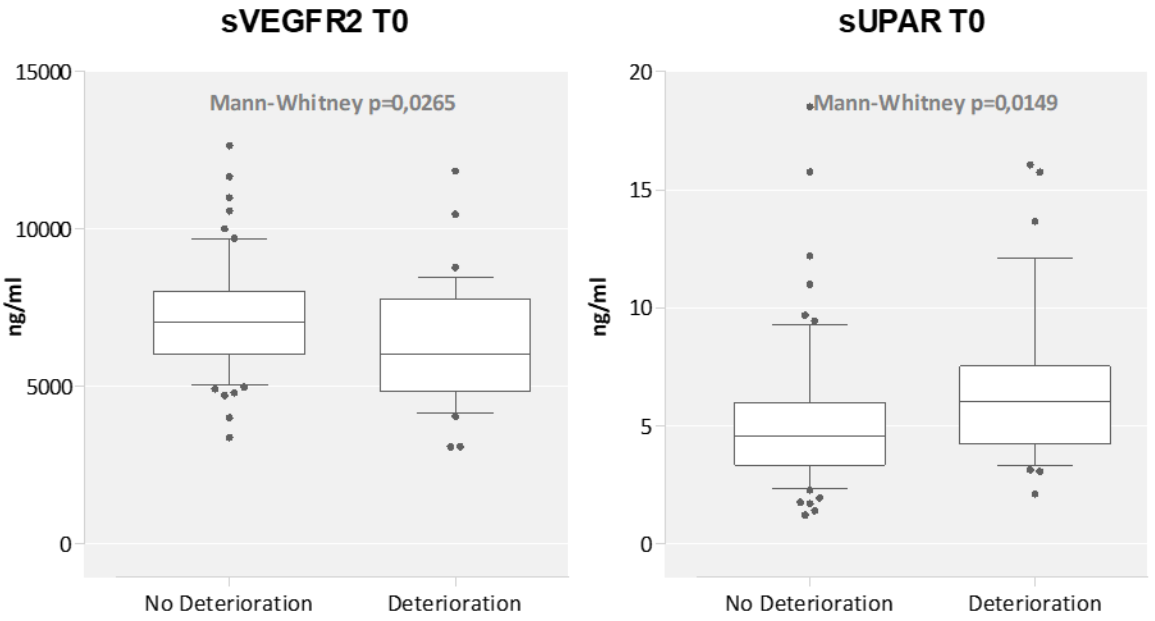

Supplemental figure 2: Matrix of deterioration representing the cumulative number of deterioration, the delta and the percentage of deterioration at each time point (A). Box plot of sVEGFR2 and sUPAR (B) proteins, between patients with or without deterioration at inclusion (H0), 6 (H6) and 24 hours later (H24). Box plot summarizes the median and interquartile ranges and P values < 0.05 are significant.

A.

TP	NbDeteriorations	Delta(T{i}; T{i-1})	%
T0	0	-	
T6	79	+79	63,7
T24	114	+35	28,2
T72	124	+10	8,1

B.

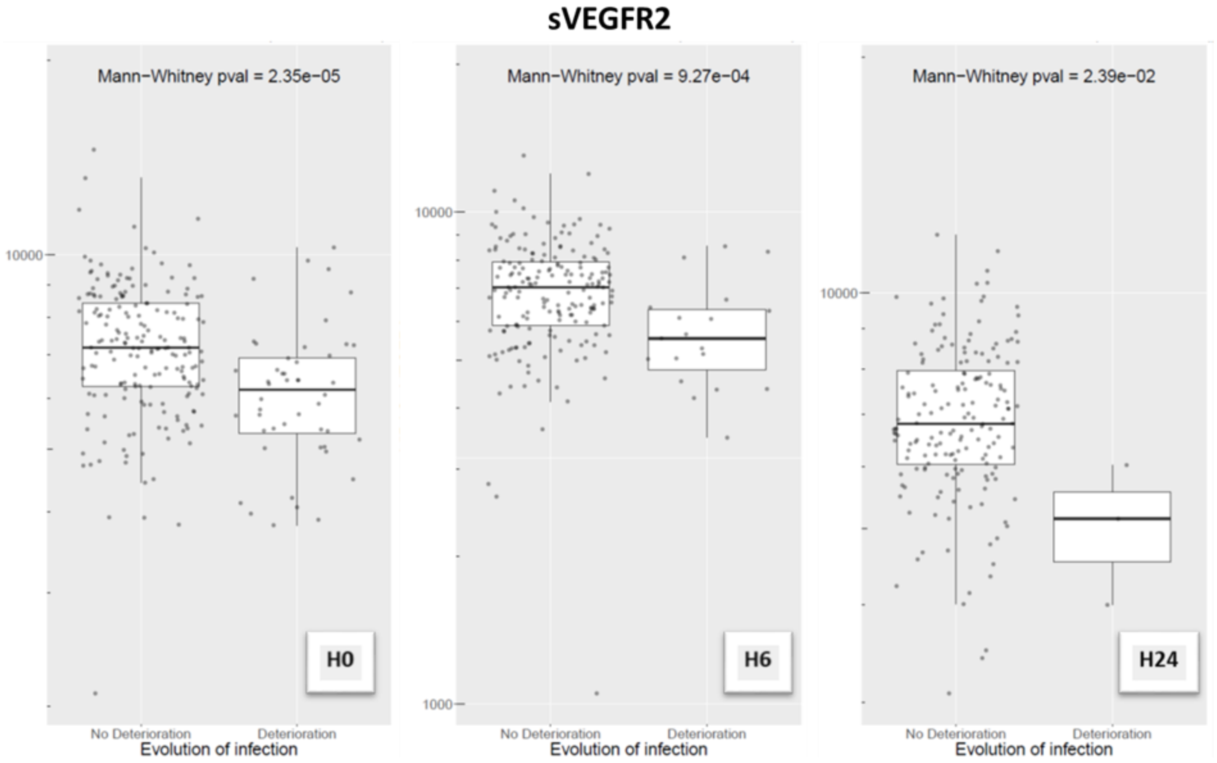

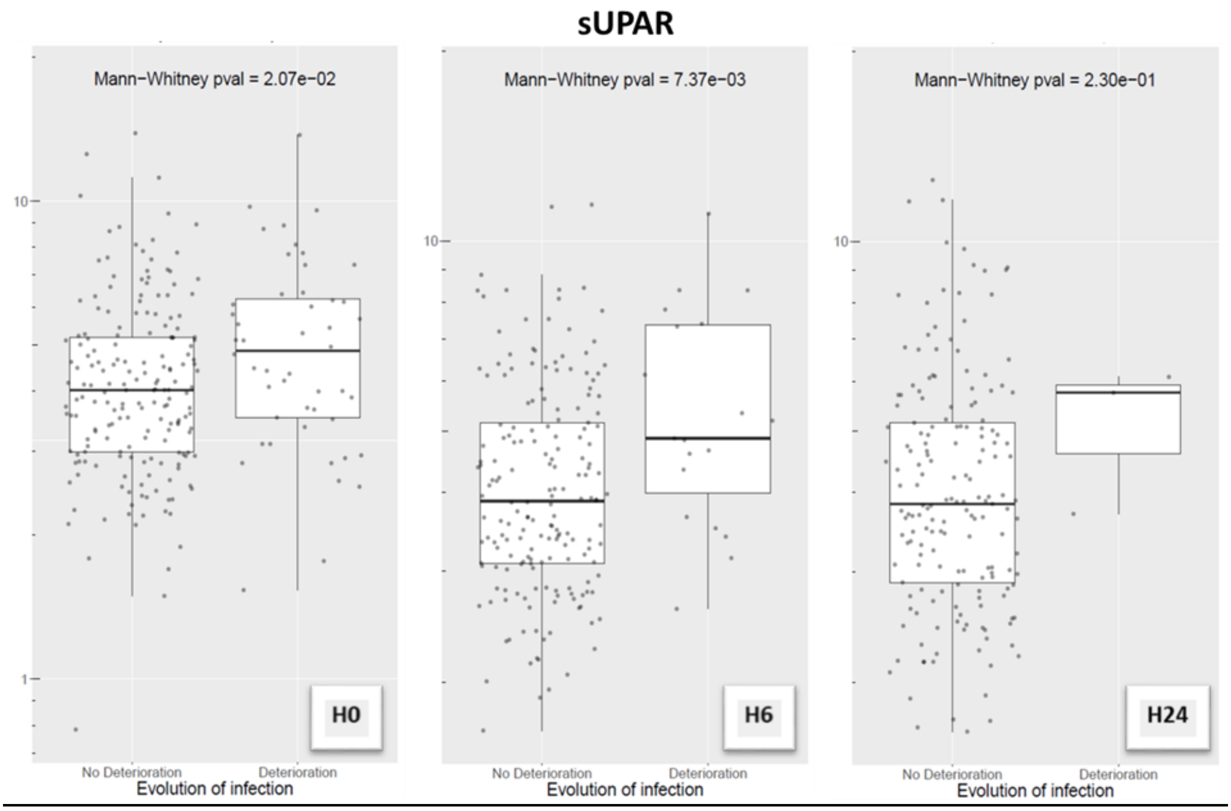
sUPAR
Mann−Whitney pval = 2.07e−02
Mann−Whitney pval = 7.37e−03
Mann−Whitney pval = 2.30e−01
10
1
H0
H6
H24
No Deterioration
Deterioration
Evolution of infection

Supplemental figure 3: Decision tree model based on threshold for sVEGFR2 and combination of both markers (sVEGFR2 and suPAR) optimised for sensitivity higher than 0.90.

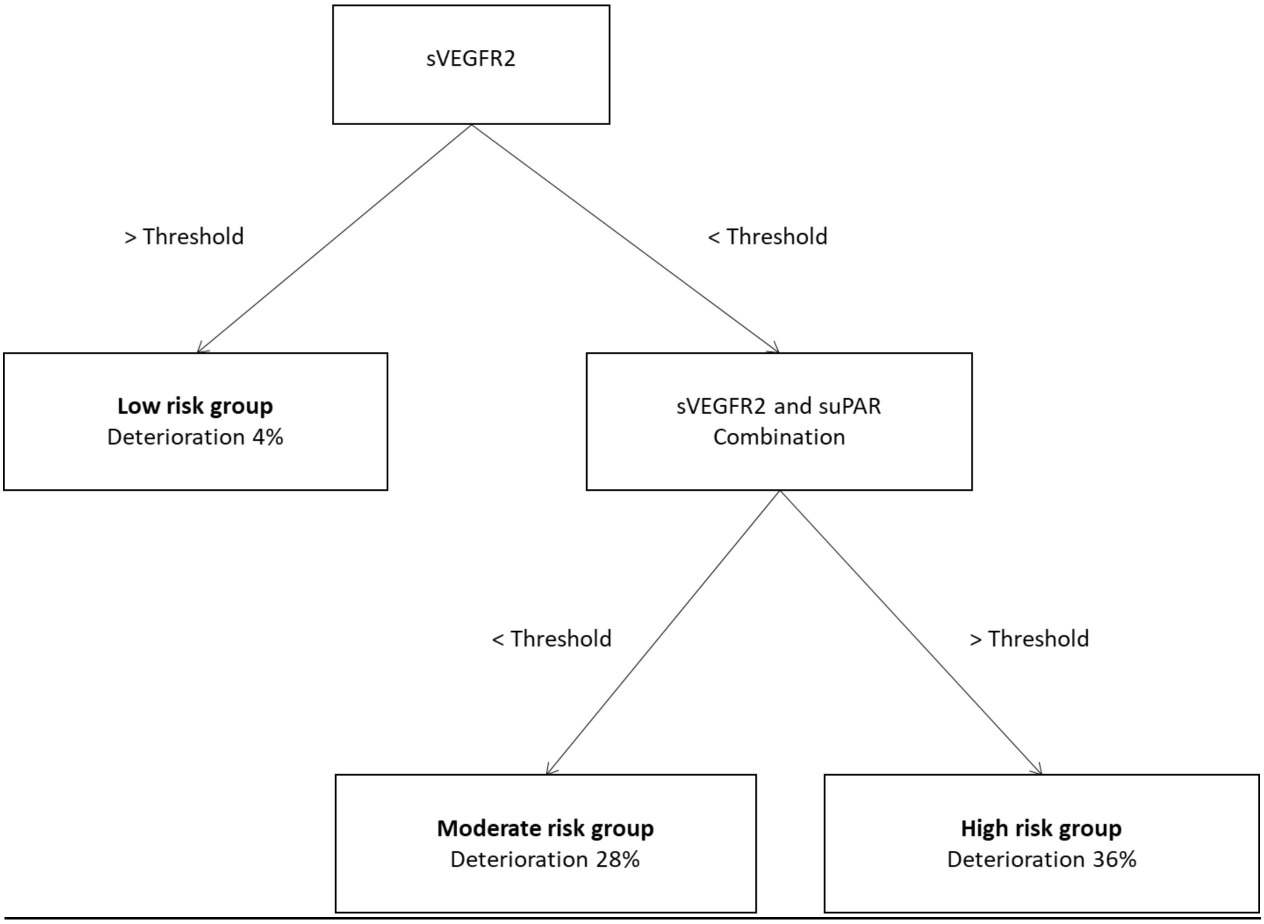

Supplemental Table 1: Factors predictive of early deterioration using univariate and multivariate analyses in septic patients (SOFA score ≥ 2) at inclusion (T0) (n=229)

	Univariate analysis		Multivariate analysis	
Variable	p-value	IQR OR [95 % CI]	p-value	IQR OR [95 % CI]
Lactates	ns	-	ns	-
CRP	ns	-	ns	-
qSOFA score	ns	-	ns	-
Age	ns	-	ns	-
SOFA total	7.94E-02		ns	
Charlson score	6.51E-02	-	ns	-
sUPAR -sVEGFR2 Combination	1.67E-05	2.06 [1.5-2.91]	1.41E-03	1.74 [1.26-2.5]

CRP: C-reactive protein: SOFA: Sequential Organ Failure Assessment; IQR: Interquartile; OR: Odds Ratio; CI: Confidence Interval
